# Strategic forecasting of renewable energy production for sustainable electricity supply: A machine learning approach considering environmental, economic, and oil factors in Türkiye

**DOI:** 10.1371/journal.pone.0328290

**Published:** 2025-08-05

**Authors:** Yasemin Ayaz Atalan, Hasan Şahin, Abdulkadir Keskin, Abdulkadir Atalan

**Affiliations:** 1 Energy Management Department, Çanakkale Onsekiz Mart University, Çanakkale, Türkiye; 2 Industrial Engineering Department, Bursa Teknik University, Bursa, Türkiye; 3 Depertmant of Statistics, Istanbul Medeniyet University, Istanbul, Türkiye; 4 Industrial Engineering Department, Çanakkale Onsekiz Mart University, Çanakkale, Türkiye; Agricultural Sciences and Natural Resources University of Khuzestan, IRAN, ISLAMIC REPUBLIC OF

## Abstract

Providing electricity needs from renewable energy sources is an important issue in the energy policies of countries. Especially changes in energy usage rates make it necessary to use renewable energy resources to be sustainable. The electricity usage rate must be estimated accurately to make reliable decisions in strategic planning and future investments in renewable energy. This study aims to accurately estimate the renewable energy production rate to meet Türkiye’s electricity needs from renewable energy sources. For this purpose, well-known Machine Learning (ML) algorithms such as Random Forest (RF), Adaptive Boosting (AB), and Gradient Boosting (GB) were utilized. In obtaining forecast data, 15 variables were considered under the oil resources, environmental parameters, and economic factors which are the main parameters affecting renewable energy usage rates. The RF algorithm performed best with the lowest mean absolute percentage error (MAPE, 0.084%), mean absolute error (MAE, 0.035), root mean square error (RMSE, 0.063), and mean squared error (MSE, 0.004) values in the test dataset. The R^2^ value of this model is 0.996% and the MAPE value is calculated lower than 10%. The AB model, on the other hand, has the highest error values in the test data set, but still provides an acceptable prediction accuracy. The R^2^ value was 0.792% and the MAPE value (0.371%) of this model was calculated to be in the range of 20% < MAPE≤50%. This study, with its proposed forecasting models, makes significant contributions to energy policies to develop appropriate policies only for planning the amount of electricity usage needed in the future. In this context, this study emphasizes that renewable energy-based electricity generation transformation should be considered as an important strategic goal in terms of both environmental sustainability and energy security.

## 1. Introduction

Renewable energy has been gaining increasing attention over the years due to the pressing need to address climate change and reduce the world’s reliance on traditional fossil fuels [1]. As the demand for electricity and oil production continues to grow, weather parameters and economic factors play crucial roles in adopting renewable energy sources. Renewable energy has been hailed as the solution to the world’s energy crisis. Unlike conventional fossil fuels such as coal, oil, and natural gas, renewable energy sources come from naturally replenishing sources, such as solar, wind, hydro, and geothermal energy [2]. These sources have a significantly lower environmental impact than traditional fossil fuels, making them a more sustainable option for meeting our energy needs. Moreover, renewable energy has the potential to boost economic growth by creating jobs, reducing reliance on imported fuels, and promoting energy independence [3].

One of the key drivers for the adoption of renewable energy sources is the increasing electricity demand. Population growth and economic growth have led to a steady increase in electricity consumption worldwide [4]. Since traditional fossil fuels are limited resources, the transition to renewable energy sources helps meet the increasing demand for electricity without depleting our planet’s resources [5]. In addition, renewable energy helps reduce dependence on imported fossil fuels, as well as reducing the impact of volatile oil prices on the economy [6,7]. Countries that are heavily dependent on imported oil are more likely to invest in renewable energy to reduce their dependence on foreign oil and hedge against oil price fluctuations. On the other hand, countries with large domestic oil reserves have less incentive to switch to renewable energy because their oil industry makes a significant contribution to their economies. Therefore, the level of oil production and imports plays an important role in shaping a country’s energy policy and adoption of renewable energy sources [8].

Renewable energy plays a crucial role in driving sustainable development and reducing dependence on fossil fuels [9]. To keep the economy growing sustainably, Türkiye has embraced various renewable energy sources such as bioenergy, solar power, hydropower, and wind power [10]. To accurately estimate renewable energy usage in Türkiye, various factors need to be considered. These factors include the amount of oil imports, weather parameters, and economic data. The research aims to assess the feasibility of different renewable energy sources in Türkiye based on various indicators such as price of electricity generation, greenhouse gas emissions, availability of renewable sources, energy conversion efficiency, land requirements, water consumption, and social impacts [11]. Based on the indicators, wind power is identified as the most sustainable energy source in Türkiye, followed by hydropower, photovoltaic, and geothermal power [12].

Environmental parameters are also important factors in the distribution of renewable energy sources [13,14]. Renewable energy systems such as solar and wind are highly dependent on weather conditions and therefore more intermittent than traditional energy sources [15,16]. The availability of sunlight and wind at any time not only significantly affects the production of these renewable energy sources, but also makes it difficult to integrate them into the electricity grid [17]. As renewable energy sources become more common, it is crucial to consider weather and other climatic variables.

Adopting renewable energy sources has both positive and negative economic impacts [18]. On the one hand, it creates jobs in the renewable energy sector, supports economic growth, and reduces the cost of electricity for consumers [19]. On the other hand, the initial investment in renewable energy infrastructure can be high, and the intermittent nature of these resources can make them less reliable, leading to potential economic consequences. For renewable energy to reach its full potential, it is crucial to carefully evaluate its economic impacts and address potential challenges [20].

The increasing availability of data and advances in machine learning (ML) have greatly impacted the renewable energy sector [21]. Data analytics has made it possible to accurately predict weather patterns and predict the output of renewable energy sources [21]. ML is also used to optimize the operation of renewable energy systems, minimizing costs and maximizing efficiency [22]. Additionally, data analysis helps policymakers make informed decisions when creating energy policies and encouraging the adoption of renewable energy sources. In the future, advances in ML algorithms and data analytics techniques will continue to improve renewable energy modeling and forecasting. With the availability of large-scale data on weather parameters, economic indicators, and oil import volumes, ML algorithms are further optimized to provide more accurate forecasts of renewable energy use [23]. These forecasts will be vital for policy and planning purposes as they can inform decision-making and help shape renewable energy strategies. Additionally, the integration of ML techniques with other data sources such as smart grid data further increases the accuracy and reliability of renewable energy forecasting [24]. A lot of studies are being done to predict renewable energy based on ML algorithms. In this study, we used ML algorithms such as Random Forest (RF), Adaptive Boosting (AB), and Gradient Boosting (GB) to estimate the rate at which renewable energy use meets electricity needs in Türkiye, taking into account factors such as oil production amounts and import price, weather parameters, and economic data. The method, data type, and regional information of some studies are given in Table 1.

**Table 1:** Studies preferring ML algorithms for forecasting in renewable energy production.

Energy Type	Country	Factors	Models	Reference
Solar	Australia	Weather	HMM, SVM regression	[25]
Wind	Spain	Time	ANN	[26]
Solar	Malaysia	Weather	SVM, GPR, LR, and DT	[27]
Wind, Solar	UK	Weather, historic power production, time	ANN, SVR, GPR	[28]
Solar Thermal Plants	Spain	Solar Radiation (hourly), Clearness index, Lost component	AR, ANN, ANFIS	[29]
PV Power	France	Solar Radiation, Time	ANN, MLP, ELM, RBF, ANFIS, ARMA	[30]
Wind	US	wind time series, simulation	k-NN, SVR	[31]
Wind	Türkiye	historical wind speed data	LASSO regression, kNN regression, xGBoost regression, RF regression, SVR	[32]
Solar, wind	Brazil, Spain	Weather, geographical	Ridge regression ensemble	[33]
solar-wind hybrid	Colombia	Environmental, Geographical	K-means	[34]
Wind, Solar, and Other	Türkiye	Environmental, Economical, Oil Resource, Population	RF, AB, GB	The present study

**Abbreviation:** HMM, Hidden Markov Model; SVM, Support Vector Machine; ANN, Artificial Neural Network; GPR, Gaussian Process Regression; LR, Linear Regression; DT, Decision Tree; SVR, Support Vector Regression; ELM, Extreme Learning Machine; RBF, Radial Basis Function; ANFIS, Adaptive Neuro-Fuzzy Inference Systems; ARMA, Autoregressive Moving Average; k-NN, k-nearest neighbors; SVR, Support Vector Regression; RF, Random Forest; LASSO, Least Absolute Shrinkage and Selection Operator; AB, Adaptive Boosting; GB, Gradient Boosting; AR, Autoregressive.

Studies have generally discussed wind and solar energy types extensively, although ML algorithms are widely used to estimate the amount of energy produced in the field of renewable energy. Li et al. tried to predict short-term solar radiation based on the Hidden Markov Model and SVM regression algorithms, which are machine learning methodologies [25]. Another study on wind energy estimated the amount of energy using the ANN algorithm from ML models [26]. SVM, GPR, LR, and DT algorithms from ML models were used to predict the PV power output of the Grid-Connected Photovoltaic System (GCPV) in Malaysia, addressing its use and contribution to society [27]. Another study used ANN, SVR, and GPR models to estimate the amount of both wind and solar energy production using weather parameters, historical production data, and periods [28]. In another study that took period as a factor, AR, ANN, and ANFIS algorithms were used to estimate the energy production obtained from solar thermal plants [29]. Fernandez-Jimenez et al. compared data obtained from two different numerical weather (NWP) forecasting programs using various statistical and AI models such as kNN, ANFIS, ARIMA, and ANN [30]. In a study, accurate prediction methods for the integration of wind power into the electricity grid were examined and it was shown that the combination of DT and SVR gave the best results and worked faster than homogeneous ensembles [31]. Another research demonstrated the usability of ML algorithms in long-term forecasts of wind energy, emphasizing that efficient predictions will be made before potential energy fields are identified [32]. Carneiro et al. brought together wind and solar energy forecasting methodologies and developed an ensemble model that aims to reduce forecast errors in different geographical locations and enable more efficient use of energy resources as a result of simulations made for solar and wind data [33]. Salazar-Caceres et al. applied the k-means model in ML algorithms to evaluate Solar-Wind hybrid energy systems based on data obtained from a local weather station [34].

One research emphasized the importance of renewable energy diversification and the role that ML algorithms can play in predicting and optimizing renewable energy use, expressing the need for policy measures and fiscal incentives to promote the adoption of renewable energy sources in Uzbekistan’s residential business [35]. Another study suggested that accurate weather forecasts and machine learning algorithms are crucial for predicting and optimizing renewable energy use [36]. Shen and colleagues demonstrated that accurate weather forecasts, economic data, and machine learning algorithms are vital to predict and optimize renewable energy use [20]. A study evaluated the feasibility of different renewable energy sources in Türkiye based on various indicators such as electricity generation price, greenhouse gas emissions, availability of renewable resources, energy conversion efficiency, land requirements, water consumption, and social environment [37]. Another study evaluated the effectiveness of using machine learning algorithms to investigate the performance difference between predicted and actual energy demand in buildings and predict building energy demand [38]

Finally, the role of policy cannot be overlooked in the promotion of renewable energy. Governments around the world have implemented various policies and regulations to encourage the deployment of renewable energy sources [39,40]. These policies include financial incentives, renewable energy goals, and portfolio standards. However, the success of renewable energy policies depends on several factors, such as political will, public acceptance, and the availability of resources. As renewable energy technology continues to evolve, policymakers must continue to adapt their policies to foster its growth. In conclusion, renewable energy is a complex and rapidly expanding sector that is influenced by several interrelated factors. The adoption of renewable energy sources is shaped by various factors, including growing electricity demand, oil production-import amounts, weather parameters, economic impacts, and policy. Achieving a more sustainable future will require a coordinated effort from governments, businesses, and consumers.

## 2. Projecting renewable energy growth and renewable energy trends in Türkiye

Renewable energy has been gaining significant momentum in recent years, with an emphasis on reducing dependence on fossil fuels and transitioning to more sustainable energy sources. For Türkiye, a country traditionally dependent on imported energy sources, this transition towards renewable energy offers tremendous opportunities for economic growth and energy security. By setting ambitious targets for renewable energy in the National Renewable Energy Action Plan (NREAP), Türkiye aims to escalate the share of renewable energy in electricity production to 30% by 2023 [41]. However, the country currently heavily relies on imported fossil fuels, with these sources making up approximately 75% of its total energy consumption [42]. This dependence on imported energy not only puts Türkiye at a disadvantage in terms of energy security but also has important economic consequences. Türkiye is actively investing in renewable energy sources, especially wind and solar energy, to reduce its dependence on imported energy. As of 2020, renewable energy sources account for approximately 45% of the total installed electricity capacity, making up the majority of wind energy, followed by hydroelectricity and solar energy [43].

Many factors have contributed to the growth of renewable energy in Türkiye, making it one of the fastest-growing markets in the region. The first and most important factor is the country’s abundance of renewable energy sources such as solar, wind, and hydroelectricity. Türkiye’s geographical location, strong winds, and high solar radiation make it an ideal candidate for wind and solar energy production [44]. In addition, the decrease in costs of renewable energy technologies played an important role in Türkiye’s growth in the sector [45]. The costs of solar and wind energy have fallen significantly over the past decade, making them more competitive with traditional energy sources. The National Renewable Energy Action Plan (NREAP) and the Renewable Energy Act of 2005 have created a stable and attractive investment environment for renewable energy projects [46]. Renewable energy in Türkiye is expected to constitute 34% of the country’s total electricity production by 2023, with wind energy making the biggest contribution to this area [47]. Türkiye is taking steps towards a sustainable and environmentally friendly energy transformation by giving increasing importance to the energy sector. The country focuses on reducing its dependence on local and renewable energy sources, as it meets most of its energy needs from abroad. In this context, Türkiye’s renewable energy transformation is considered an important strategic goal in terms of both environmental sustainability and energy security.

Türkiye’s geographical location, especially the Aegean and Marmara regions, has suitable conditions for wind energy projects [48]. In recent years, there has been a global shift towards renewable energy sources to address growing concerns such as climate change and fossil fuel depletion [49]. Türkiye, a developing country with high energy demand, has also realized the importance of transitioning to renewable energy sources. Türkiye’s energy sector is heavily dependent on imported fossil fuels, making it vulnerable to price fluctuations and supply disruptions. In 2019, fossil fuels accounted for 86% of the country’s total energy consumption, with natural gas making the largest contribution at 31%, followed by coal at 28% [50]. On the other hand, renewable energy sources accounted for only 14% of the energy mix, with hydropower being the dominant source at 12% [51]. However, Türkiye has made significant progress in recent years in increasing its renewable energy capacity. According to the International Renewable Energy Agency (IRENA), Türkiye’s renewable energy capacity increased by 11.6% in 2019, with solar and wind energy contributing the most to this growth [52].

The country aims to reach a 30% share of renewable energy in electricity generation by 2023 and a 50% share by 2050. To achieve these targets, Türkiye has implemented various policies and incentives to promote renewable energy deployment. One of the main drivers for renewable energy growth in Türkiye is the Renewable Energy Support Scheme (YEKDEM), which was introduced in 2011. Under this scheme, renewable energy producers are guaranteed a fixed price for their electricity for 10 years. This has encouraged private investors to enter the renewable energy market and has resulted in significant growth in solar and wind energy installations. In addition, Türkiye also introduced net metering regulations in 2016, allowing consumers to generate their electricity from renewable sources and sell excess energy back to the grid. This has led to an increase in rooftop solar installations, especially in the residential and commercial sectors.

The rest of the study is organized as follows: the data and methodology of the study are described in the third section. The fourth part of the study includes the results. This section also includes discussion and policy implications and recommendations parts. The last section consists of a conclusion and suggestions for future studies.

## 3. Methodology

In this study, Machine Learning (ML) algorithms Random Forest (RF), Adaptive Boosting (AB), and Gradient Boosting (GB) models were run to estimate the ratio of renewable energy to electricity production, taking into account the oil [53], environmental [54], and economic [55] input parameters that are effective in obtaining the consumed electricity from renewable energy sources [56].

### 3.1 Renewable energy sources in gross electricity consumption in Türkiye

**Fig 1:**
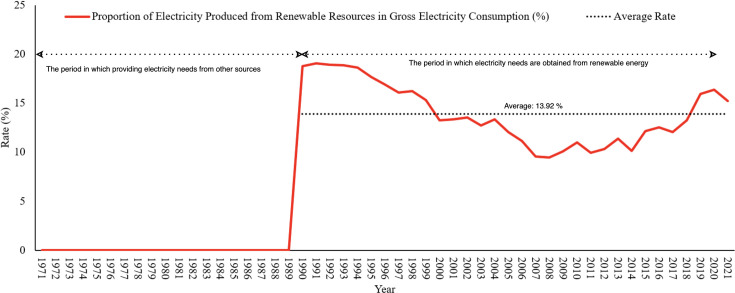
Rates (%) of electricity produced from renewable energy sources in gross electricity consumption.

In this study, data on Türkiye’s electricity needs from renewable energy sources were used. Türkiye started to use renewable energy sources for its electricity needs in 1990. However, in this study, data on the amount of energy produced from renewable energy sources covering the years 1971–2021 was used to consider the effect of renewable energy sources on electricity use and to see the effect of forecast data only on the rate of electricity usage from renewable energy types, and these data are shown in Fig 1.

**Fig 2:**
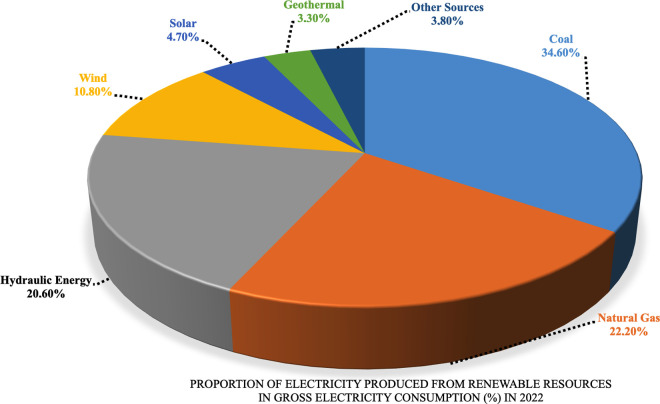
The proportion of electricity produced from all energy resources in Türkiye’s gross electricity consumption (%) in 2022.

When the ratio of electricity produced from renewable sources to total electricity consumption in Türkiye in 2022 is examined, it is seen that coal has the largest share with 34.6%. Then, natural gas ranks second with 22.2%, while hydraulic energy ranks third with 20.6%. Wind energy comes in fourth place with a share of 10.8%, while solar energy follows it with 4.7%. Geothermal energy is among other renewable resources at 3.3%, while other resources have a rate of 3.8%. Approximately 15.5% of electricity needs were obtained from renewable energy sources. Rates (%) of electricity produced from all energy sources in Türkiye’s gross electricity consumption in 2022 are shown in Fig 2.

In this study aimed at meeting electricity consumption from renewable energy sources, population, environmental, economic, and oil factors are discussed under the main headings as input parameters. Some sub-factors of environmental, economic, and petroleum factors were taken into account and the detailed effects of these factors on renewable energy were considered.

### 3.2 Oil production and barrel price of imported oil

**Fig 3:**
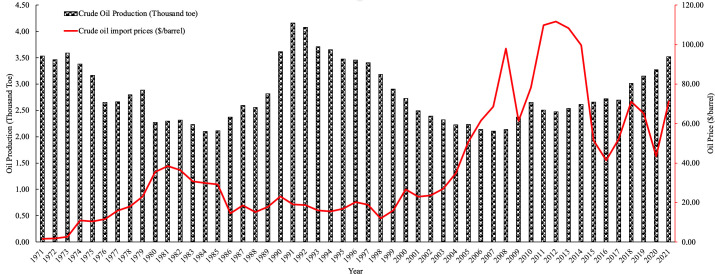
The amount of crude oil production and crude oil import price.

Basing electricity consumption on renewable energy sources has a strategic importance in terms of reducing oil production and imports. Electricity from traditional energy sources is generally based on fossil fuels, especially oil-based energy sources. This situation causes the energy sector to be highly dependent on oil demand, leading to energy security risks. The use of renewable energy sources both reduces this dependence and allows energy supply to be met in a more diverse, sustainable, and independent manner. Moreover, considering the strategic importance and geopolitical effects of oil, the use of renewable energy increases national security and supports environmental sustainability by reducing dependence on energy imports. For this reason, in this study, data on Türkiye’s annual oil production amount and the price per barrel of imported oil are treated as independent variables. Data on meeting Türkiye’s electricity needs from oil resources, the amount of oil produced for electricity production, and the barrel price of imported oil are shared in Fig 3.

### 3.3 Environmental parameters

Environmental parameters play a very important role in renewable energy forecasts. Accurate and reliable weather forecasts are necessary to optimize the use of weather-dependent renewable energy sources such as wind, hydropower, and solar power. In particular, by analyzing historical weather data to obtain forecast data and correlating it with renewable energy production, patterns and relationships between weather parameters and renewable energy production are determined. Algorithms such as machine learning take into account factors such as wind speed, solar radiation, precipitation, and temperature to predict renewable energy production levels (Zeng et al., 2016). In this study, taking into account the factors of air temperature (^0^C), Wind Speed (m/sec), Air Pressure (hPa), Relative Humidity (%), Sunshine Time (hr), and total annual precipitation amount (mm), which are among the weather parameters. Their effects on renewable energy production were analyzed.

### 3.4 Economic parameters

Economic parameters such as GDP, GDPC (gross domestic product per capita), household expenditures, currency, and inflation, which are among the economic parameters that have an impact on renewable energy projects in terms of generating electricity from renewable energies, were taken into account in this study. Renewable energy projects often require large-scale infrastructure investments, which affects the economic growth potential of countries. Successful implementation of these projects has the potential to create jobs, advance technology, and provide overall economic revival. GDPC represents the economic well-being of individuals, and the use of renewable energy sources generally supports a sustainable and green model of economic development. In addition to household expenditures being sensitive to changes in energy costs, the decrease in the cost of renewable energy sources provides economic relief to consumers by offering lower energy bills. Additionally, currency and inflation factors have a significant impact on the competitiveness of renewable energy investments by affecting energy costs and financing of projects. Therefore, consideration of economic parameters plays a critical role in the successful implementation of renewable energy projects and in determining strategies for sustainable energy transition.

### 3.5 Machine learning (ML) algorithms

Machine learning (ML) undertakes the function of providing computer systems with the ability to analyze data and learn from this data. This technology is known for its ability to identify patterns in complex data sets, create predictable patterns, and predict future events. ML algorithms learn using labeled or unlabeled data and continuously improve through this learning process. In classification tasks, an ML model separates data into different categories based on a specific input feature. Likewise, in regression tasks, ML models predict future values by analyzing the relationship between input variables and output variables. These functions in ML are used to power data-driven decision-making in energy, marketing, finance, healthcare, transportation, and many more industries.

**Fig 4:**
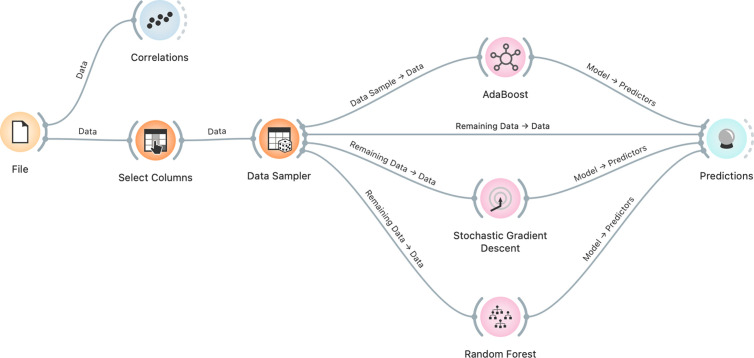
The overview of the ML algorithms’ flowchart.

Random Forest (RF), Adaptive Boosting (AB), and Gradient Boosting (GB) are three popular ensemble learning techniques used in ML to improve the predictive performance of individual models. Ensemble learning techniques involve combining multiple base learners to create a stronger, more accurate model. In this paper, we will discuss the concepts, benefits, and applications of RF, AB, and GB, as well as how they differ from each other. These three ML algorithms, RF, AB, and GB, are particularly useful for estimating renewable energy usage in Türkiye based on the given factors due to their ability to handle multiple variables, detect patterns in complex data, and provide accurate predictions. The machine learning model used in this study is shown in Fig 4.

#### 3.5.1 Random Forest (RF).

Random Forest (RF) combines multiple decision trees to create a powerful predictive model [57]. The algorithm randomly selects a subset of features and a subset of training data to build each decision tree [58]. This situation helps to reduce the variance in the predictions and avoid overfitting, resulting in a more robust model [59]. Additionally, RF performs feature selection, where it ranks the importance of each feature in predicting the target variable, making it easier to interpret the results [60]. In the RF algorithm, by generating random vectors, each of the $\varphi_{t}$, which is a subset of the feature space of the dataset, is created using the $\varphi_{t}$ and the training dataset. The generalization error and margin function in the RF are described in Eq [1] and [2] [61]:


$$P_{X,Y}(mg(X,Y)<0)$$ (1)

Here,


$$\begin{array}{c} mg(X,Y)=\alpha s_{t}I\left(D_{t}\left(V_{1}\right)=V_{2}\right)-\max{\mathrm{\backslash nolimits}}_{j\neq Y}{\alpha s}_{t}I\left(D_{t}\left(V_{1}\right)=j\right) \end{array}$$ (2)

where random vectors are defined as $V_{1}$ and $V_{2}$. mg is assigned as the margin function used when comparing the correct output with other outputs by checking the average votes in a random vector. The indicator function is defined as $I(D_{t}\left(V_{1}\right)=V_{2}and\;I\left(D_{t}\left(V_{1}\right)=j\right)$, and hk refers to the classifiers. The operating parameters of the RF model preferred for this study are given in Table 2.

**Table 2:** Parameters of the RF Model.

Name	Random Forest
**Tree Numbers**	10
**Number of features**	unlimited
**Repeatability of training data**	No
**The tree depth**	unlimited
**Split nodes by maximum samples (stop)**	5
**Features**	Crude Oil Production (Thousand toe), Population (Million persons), Crude oil import prices ($/barrel), GDP PC ($), GDP (Million $), Currency (TL/$), Inflation (%), Household spending (Million $), Temp (C), Wind Speed (m/sec), Air Pressure (hPa), Relative Humidity (%), Sunshine Time (Hr), Precipitation (Total, mm), Average Rate (total: 15 features)
**Meta attributes**	Year
**Target**	The proportion of Electricity Produced from Renewable Resources in Gross Electricity Consumption (%)

#### 3.5.2 Adaptive Boosting (or AdaBoost) (AB).

Adaptive Boosting (AB), also known as AdaBoost, is another popular ensemble learning technique that works by combining weak learners to create a strong predictive model [62]. Initially, all the data points are given equal weights, and a base model is trained. In the next iteration, the weights of the misclassified data points are increased, and the model is trained again. This process is repeated multiple times, and the final model is created by combining the predictions of all the weak learners. AB focuses on the difficult data points and assigns them more weight, making it more robust to outliers and noisy data [63]. The AB algorithm is used in Eq. [3] to linearly combine weak classifiers to create a strong classifier [64]:


$$F(x)=\underset{c}{\underbrace{max}}\sum_{l=1}^{L}w_{l}(v)=\underset{c}{\underbrace{max}}\sum_{l=1}^{L}w(v,\;f_{t},\;\varphi_{l},\;c)$$ (3)

where, $w_{l}$ represents the weak learner at iteration l, $\varphi_{l}$ signifies a threshold value, v denotes a feature vector within the PCA space, and $f_{t}$ indicates that the f^th^ component of v is utilized as an input feature in weak learner $w_{l}(v)$. Algorithm 1.1. stated below was used for the AB algorithm [65].

***Algorithm 1.1. Adaptive Boosting Model***

Initialize observation weights $w_{l}=\frac{1}{M}$

**For** l=1 to L **do**

Fit $f_{t}\left(x\right)$ as the weak classifier on the training dataset using $w_{l}$

compute the weighted error rate as $\varepsilon_{l}=\frac{\sum_{l=1}^{L}w_{l}*I(y_{i},\neq f_{l}(x_{l}))}{\sum_{l=1}^{L}w_{l}}$

Let $\alpha_{l}=log(\frac{1-\varepsilon_{l}}{\varepsilon_{l}})$

Revised $w_{l}\leftarrow w_{l}*e[\alpha_{l}*I\left(y_{i}\neq f_{l}\left(x_{l}\right)\right)]$ scaled to sum to one ∀ l∈{1, …, L}

**End for**

Output $\hat{f}(x)=sign[\sum_{l=1}^{L}a_{l}*\hat{f_{l}}(x)$

The operating parameters of the AB model preferred for this study are given in Table 3.

**Table 3:** Parameters of AB Model.

Name	Gradient Boosting (Scikit-learn)
**Estimator Type**	Tree
**Estimator Numbers**	50
**Type of classification**	Samme.r
**Loss (regression type)**	Linear
**Features**	Crude Oil Production (Thousand toe), Population (Million persons), Crude oil import prices ($/barrel), GDP PC ($), GDP (Million $), Currency (TL/$), Inflation (%), Household spending (Million $), Temp (C), Wind Speed (m/sec), Air Pressure (hPa), Relative Humidity (%), Sunshine Time (Hr), Precipitation (Total, mm), Average Rate (total: 15 features)
**Meta attributes**	Year
**Target**	The proportion of Electricity Produced from Renewable Resources in Gross Electricity Consumption (%)

Predictive data was obtained by selecting samme.r classification for the AB algorithm. The Samme.r algorithm of the AB model is given below as **Algorithm 1.2** [59,66].

***Algorithm 1.2. SAMME.R algorithm***

*Step 1. Initialize the observation weights* $w_{i}=\frac{1}{n},\;i=1,2,\ldots ,\;n.$

*Step 2. For* m=1 to M*:*

*Step 2.1. fit a classifier* $T^{\left(m\right)}(x)$ *to the training data using weights* $w_{i}$*.*

*Step 2.2. obtain the weighted class probability estimates:*

$P_{k}^{\left(m\right)}(x)={Prob}_{w}(c=k\setminus x),\;k=1,\;2,\;\ldots ,\;K$

*Step 2.3. set:*

$h_{k}^{\left(m\right)}(x)\leftarrow (K-1)(logp_{k}^{m}(x)-1/K\sum_{k^{\prime }}\log p_{k^{\prime }}^{\left(m\right)}(x)),\;k=1,\;2,\;\ldots ,\;K$.

*Step 2.4. set:*

$w_{i}\leftarrow w_{i}.exp\;(-\frac{K-1}{K}y_{i}^{T}\log p^{\left(m\right)}\left(x_{i}\right),\;i=1,\;2,\;\ldots ,\;n$.

*Step 2.5. re-normalize* $w_{i}$*.*

*Step 3. Output*

           C(x)=arg(maxk)∑m=1Mhk(m)(x)

where, the input variable is labeled as $x_{i}$, and the response variable value is represented as c within a finite set, both defined for the training dataset. $w_{i}$ represents the weight coefficient of the algorithm. the weighted class probability is defined as $P_{k}^{\left(m\right)}(x)$. The misclassification error rate, denoted as $C_{\left(x\right)}$, is calculated. Optimal model performance is achieved when this rate is minimized. A weak multi-class classifier is denoted as as T(x). An improved estimate $h_{k}^{\left(m\right)}(x)$ is obtained by minimizing the loss for each x.

#### 3.5.3 Gradient Boosting (GB).

Gradient Boosting (GB) is an ensemble learning technique that works by sequentially combining multiple weak learners [67]. Unlike AB, it uses a more sophisticated approach where each subsequent model learns from the errors of the previous model. In each iteration, the algorithm calculates the residual error of the model and uses it as the target variable for the next model. This process continues until the algorithm reaches a predefined number of iterations or until the error cannot be reduced any further. This results in a model that is highly tuned to the data and can capture complex relationships between the features and the target variable. Algorithm 2. stated below was used for the AB algorithm [65].

**Algorithm 2. Gradient Boosting Model**

Initialize observation weights $f_{0}(x)$ to be a constant, $f_{0}(x)=arg\min_{\gamma }\sum_{l=1}^{L}L(y_{i},\;\gamma )$

**For** l=1 to L **do**

Compute the negative gradient as the running output $r_{l}={\left[\frac{\partial L\left(y_{i},\;f\left(x_{i}\right)\right)}{\partial f\left(x_{\dot{\text{I}}}\right)}\right]}_{f(x)=f_{l-1(x)},\;i=\{1,\;\ldots ,\;N\}}$

Fit a regression model to $r_{i}$ by the leat-squares using the input $x_{i}$ and get the estiamte $a_{t}$ of γh(x;α)

Get the estimate $\gamma_{l}$ by minimizing $L(y_{i},\;f_{l-1\left(x_{i}\right)}+\gamma h(x_{i};\alpha_{l})$

Revised $f_{l}(x)=f_{l-1}(x)+\gamma_{l}h(x;\alpha_{l})$

**End for**

Output $\hat{f}(x)=f_{L}(x)$

The operating parameters of the GB model preferred for this study are given in Table 4.

**Table 4:** Parameters of RF Model.

Name	Gradient Boosting (Scikit-learn)
**Tree numbers**	100
**Rate of Learning**	0.1
**Repeatability of training data**	Yes
**Tree depth**	3
**Split nodes by maximum samples (to stop)**	5
**Proportion of training samples**	1
**Features**	Crude Oil Production (Thousand toe), Population (Million persons), Crude oil import prices ($/barrel), GDP PC ($), GDP (Million $), Currency (TL/$), Inflation (%), Household spending (Million $), Temp (C), Wind Speed (m/sec), Air Pressure (hPa), Relative Humidity (%), Sunshine Time (Hr), Precipitation (Total, mm), Average Rate (total: 15 features)
**Meta attributes**	Year
**Target**	Proportion of Electricity Produced from Renewable Resources in Gross Electricity Consumption (%)

#### 3.5.4 The performance criteria of ML models.

Five performance criteria are used to evaluate the accuracy of ML models. Each of them measures the success of the ML model from different perspectives, and by selecting an appropriate measurement metric, the performance of the model is analyzed. In the rest of this section, information about the performance criteria is given respectively.

RMSE (Root Mean Squared Error) is the square root of the mean of the squares of the differences between actual and predicted values. This metric shows how much the model’s predictions deviate from the actual values. A lower RMSE indicates that the model performs better. The formula for the RMSE criterion is given in Eq [4]:


$$\begin{array}{c} RMSE=\sqrt{\frac{1}{n}\sum_{i=1}^{n}{\left(y_{i}-{\hat{y}}_{i}\right)}^{2}} \end{array}$$ (4)

where n is the number of data points, $y_{i}$ represents the actual values, and ${\hat{y}}_{i}$ represents the predicted values.

MSE (Mean Squared Error) is the mean square of the differences between actual and predicted values. It can also be thought of as the square of the RMSE. MSE also shows how much predictions deviate from actual values, but it is not as directly interpretable as RMSE. The MSE formula is expressed in Eq [5]:


$$\begin{array}{c} MSE=\;\frac{1}{n}\sum_{i=1}^{n}{\left(y_{i}-{\hat{y}}_{i}\right)}^{2} \end{array}$$ (5)

MAE (Mean Absolute Error) is the average of the absolute differences between actual and predicted values. This metric shows how much the model’s predictions deviate from actual values, but reduces the impact of large deviations by taking the absolute values of the differences. The formula expressing MAE is shown in Eq [6]:


$$MAE=\;\frac{1}{n}\sum_{i=1}^{n}\left|y_{i}-{\hat{y}}_{i}\right|$$ (6)

MAPE (Mean Absolute Percentage Error) is the average of the absolute percentage differences between actual and predicted values. This metric shows how much error, as a percentage, the model’s predictions make relative to the actual values. MAPE is especially useful when you want to understand the error rate in percent. The MAE formula is given in Eq [7]:


$$MAPE=\;\frac{1}{n}\sum_{i=1}^{n}\left|\frac{y_{i}-{\hat{y}}_{i}}{y_{i}}\right|*100$$ (7)

MAPE value is generally evaluated in four parts: MAPE<10% provides high predictive values, 10% < MAPE≤20% provides good predictive values, 20% < MAPE≤50% provides acceptable and MAPE>50% provides poor predictive values [68].

R^2^ (R-squared, Coefficient of Determination) indicates how much the model explains the variance of the independent variables by the variance of the dependent variable. An R^2^ value close to 1 indicates that the model explains most of the variability in the data set, while a value close to 0 indicates that the model explains very little of the variability in the data set. A negative R^2^ value indicates that the model performed worse than expected. The formula for R^2^ is given in Eq [8]:


$$R^{2}=\;1-\frac{\sum_{i=1}^{n}{\left(y_{i}-{\hat{y}}_{i}\right)}^{2}}{\sum_{i=1}^{n}{\left(y_{i}-{\overset{―}{y}}_{i}\right)}^{2}}$$ (8)

where ${\overset{―}{y}}_{i}$ is considered as the average of the actual values.

### 3.6 TOPSIS for ranking ML algorithms based on their performance

One of the Multi-criteria decision making (MCDM) methods, known as technique for order preference by similarity (TOPSIS), considers each alternative’s distance from the positive ideal solution (PIS) as well as its distance from the negative ideal solution (NIS). That is, the optimal alternative should be closest to the PIS and furthest from the NIS. In other words, the best option is the alternative with the shortest distance to the PIS and the longest distance to the NIS. In this study, the following equations were applied for TOPSIS ranking using the performance values of ML algorithms. To normalize the decision matrix, Eq. [9] have been used [69]:


$$y_{ij}=\frac{x_{ij}}{\sqrt{\sum_{i=1}^{m}x_{ij}^{2}}};\;i=1,\ldots ,\;m\;;j=1,\;\ldots \;,\;n$$ (9)

where $x_{ij}$ is a value corresponding to each feature of the decision maker in the decision matrix. Normalized performance values are expressed as $x_{ij}$. According to the formula below, the normalized matrix is multiplied by the criteria weights [70]:


$$v_{ij}=w_{j}y_{ij};\;i=1,\ldots ,\;m\;;j=1,\;\ldots \;,\;n$$ (10)

where, normalized $\left(y_{ij}\right)$ and weighted $(w_{j}$performance rating is denoted by $v_{ij}$. In this study, the weighted value of the j-th ctiteria’s total criteria is considered as 1. The goal of the TOPSIS method is to determine the distance of each alternative to the positive $\left(A^{+}\right)$ and negative ${(A}^{-})$ ideal points. Therefore, at this stage, $A^{+}$ and $A^{-}$ are determined by the following formulas [71]:


$$A^{+}=\left(v_{1}^{+},v_{2}^{+},\;\ldots ,\;v_{n}^{+}\right)$$ (11)

$$A^{-}=(v_{1}^{-},v_{2}^{-},\;\ldots ,\;v_{n}^{-+})$$ (12)

Here,


$$v_{j}^{+}=\left\{\begin{array}{c} \left(\max{\;v}_{ij}\right|\;j\epsilon j_{1}\;\\ \left(\min{\;v}_{ij}\right|\;j\epsilon j_{2})\; \end{array}\right\}\;i=1,\ldots ,\;m$$ (13)

$$v_{j}^{-}=\left\{\begin{array}{c} \left(min\;v_{ij}\right|\;j\epsilon j_{1})\\ \left(\max{\;v}_{ij}\right|\;j\epsilon j_{2}) \end{array}\right\}\;i=1,\ldots ,\;m$$ (14)

where, the symbols $j_{1}$ and $j_{2}$ represent the negative and positive criteria, respectively. The TOPSIS method evaluates the position of each option by considering its proximity to the PIS and its distance from the NIS. Hence, during this stage, the distances between each option and the positive $\left(d_{i}^{+}\right)$ and negative ${(d}_{i}^{-})$ ideal solutions are computed using the using the provided Eq. [14] and [15] [72,73]:


$$\begin{array}{c} d_{i}^{+}=\sqrt{\sum {\mathrm{\backslash nolimits}}_{j=1}^{n}{\left[v_{ij}-v_{j}^{+}\right]}^{2}}\;,\;i=1,\;\ldots ,\;m \end{array}$$ (15)

$$\begin{array}{c} d_{i}^{-}=\sqrt{\sum {\mathrm{\backslash nolimits}}_{j=1}^{n}{\left[v_{ij}-v_{j}^{-}\right]}^{2}}\;,\;i=1,\;\ldots ,\;m \end{array}$$ (16)

During this phase, the score of relative proximity $S_{i}$ of each option to the optimal solution is determined using the Eq. [17] [74].


$$S_{i}=\frac{d_{i}^{-}}{\left(d_{i}^{+}+d_{i}^{-}\right)}\;,\;i=1,\;\ldots ,\;m$$ (17)

If the $S_{i}$ approaches 1.00, it indicates that the option has a shorter distance from the PIS and a longer distance from the NIS. The alternatives considered are ranked based $S_{i}$ scores.

## 4. Results

The data obtained for this study were evaluated in three stages. First of all, descriptive statistics of the data were calculated. In the second stage, the performance values of ML models were evaluated. Finally, the predicted values obtained from the ML models preferred for this study were compared with the real data.

### 4.1 Descriptive statistical analysis of variables

In this study, a total of 15 variables were taken into account, 14 of which were independent (under the main headings of population, economy, environment, and oil) and 1 dependent (Proportion of Electricity Produced from Renewable Resources in Gross Electricity Consumption (%)). Class, type, mean, standart deviation, variance minimum, maximum, skewness, and kurtosis values included in the descriptive statistics of each variable were calculated. Descriptive statistics data for the variables that make up the data set of the study are given in Table 5.

**Table 5:** The value of variables based on descriptive statistics analysis.

Variable	Class	Type	Mean	StDev	Var	Min	Max	Skew	Kurt
Population (Million persons)	Population	Input	59.3	14.17	200.8	35.32	83.38	−0.03	−1.15
Crude Oil Production (Thousand	Oil	Input	28,307	0.5565	0.3097	20,978	41,589	0.59	−0.67
Crude oil import prices ($/barrel)		Input	37.59	29.76	885.69	1.7	111.7	1.17	0.48
GDP PC ($)	Economic	Input	11.22	8.06	65.02	1.77	28.68	0.98	−0.13
GDP (Million $)		Input	0.7694	0.6938	0.4814	0.0626	23,913	1.16	0.2
Currency (TL/$)		Input	0.984	1,550	2,402	0	7,009	2.17	5.12
Inflation (%)		Input	36.71	28.82	830.62	6.25	105.22	0.67	−0.81
Household spending (Million $)		Input	0.3775	0.3402	0.1158	0.0291	11,475	1.03	−0.14
Temperature (C)	Environment	Input	7,859	0.805	0.647	6,000	10,000	0.5	0.36
Wind Speed (m/sec)		Input	5,208	1,083	1,172	2,063	7,050	−0.49	0.06
Air Pressure (hPa)		Input	818.41	1.11	1.24	816.53	820.76	0.39	−0.59
Relative Humidity (%)		Input	63,592	1,505	2,266	59,600	66,700	−0.29	0.27
Sunshine Time (Hr)		Input	7.72	7.61	57.99	6	61	7.13	50.87
Precipitation (Total. mm)		Input	621.35	69.29	4800.57	493.1	793.8	0.12	−0.29
Proportion of Electricity Produced from Renewable Resources in Gross Electricity Consumption (%)	Energy	Output	8.73	7.23	52.24	0	19.05	−0.18	−1.59

**Abbreviation:** StDev, Standard Deviation; Var, Variance; Min, Minimum value; Max, Maximum value; Skew, Skewness; Kurt, Kurtosis.

The Population (Million persons) variable indicates the population for 51 years in million people. According to these data, the average value of the population is 59.3 million people and its standard deviation is 14.17 million people. However, the minimum population is 35.32 million people while the maximum population is 83.38 million people. This variable is defined as an independent variable.

Descriptive statistical data of two independent variables were calculated under the main heading of oil, which is effective in the production of renewable energy. Crude Oil Production (Thousand) Crude oil production for 51 years is stated as thousand tons. Average crude oil production was calculated as 28,307 thousand tons and its standard deviation was 0.5565 thousand tons. While the minimum production amount is 20,978 thousand tons, the maximum production amount is stated as 41,589 thousand tons. Under the heading Crude oil import prices ($/bar), crude oil import prices are stated in dollars. The average price was calculated as 37.59 dollars/barrel and the standard deviation was 29.76 dollars/barrel. While the minimum price is 1.7 dollars/barrel, the maximum price is stated as 111.7 dollars/barrel.

Descriptive statistical data for five different indicators under the heading of Economy were calculated. First of all, gross domestic product (GDP) per capita is expressed in dollars. The mean GDP per capita is $11.22 and its standard deviation is $8.06. The lowest value of GDP per capita is $1.77, while the highest value is $28.68. Second, Gross Domestic Product (GDP) is expressed in millions of dollars. The mean GDP value is $0.7694 million and its standard deviation is $0.6938 million. The lowest GDP value is 0.0626 million dollars, while the highest value is 23,913 million dollars. The third indicator, Currency (TL/$), expresses the value of Turkish Lira against the Dollar. The average exchange rate is 0.984 TL/$ and its standard deviation is 1550 TL/$. The highest exchange rate between 1971 and 2021 was 7,009 TL/$. Another economic indicator, the inflation rate, was taken into account in this study. The average inflation rate is 36.71% and its standard deviation is 28.82%. While the lowest inflation rate is 6.25%, the highest value is 105.22%. Finally, the Household spending (Million $) indicator is treated as an independent variable. Mean household expenditures are $0.3775 million with a standard deviation of $0.3402 million. The lowest spend is $0.0291 million, while the highest spend is $11,475 million.

Descriptive statistical data for six different indicators under the title of environment that are effective in renewable energy production were calculated. The mean temperature is 7.859°C with a standard deviation of 0.805°C. The lowest temperature is 6°C, while the highest temperature is 10°C. The average wind speed is 5,208 m/s and its standard deviation is 1,083 m/s. The lowest wind speed is 2,063 m/s, while the highest wind speed is 7,050 m/s. The mean air pressure is 818.41 hPa and its standard deviation is 1.11 hPa. The lowest air pressure is 816.53 hPa, while the highest air pressure is 820.76 hPa. The average relative humidity is 63.592% and its standard deviation is 1.505%. The lowest relative humidity is 59,600%, while the highest relative humidity is 66,700%. The average sunshine duration is 7.72 hours and its standard deviation is 7.61 hours. While the lowest sunshine duration is 6 hours, the highest sunshine duration is 61 hours. The average rainfall is 621.35 mm and its standard deviation is 69.29 mm. While the lowest rainfall amount is 493.1 mm, the highest rainfall amount is 793.8 mm.

Finally, the output variable is the Proportion of Electricity Produced from Renewable Resources in Gross Electricity Consumption (%). The mean rate is 8.73% and its standard deviation is 7.23%. While the lowest rate is 0% (values taken into account for periods when renewable energy production does not affect electricity use), the highest rate is 19.05%.

Table 6 presents correlation values that express the relationships between different variables. A positive correlation indicates a linear relationship between two variables, where an increase in one variable results in an increase in the other, while a negative correlation indicates that an increase in one variable results in a decrease in the other. Correlation coefficients range from −1 to +1. A value closer to −1 indicates a stronger negative relationship, while a value closer to +1 indicates a stronger positive relationship. A value of 0 indicates no relationship between the two variables.

**Table 6:** The correlation values of variables.

Variable	Year	Population (Million persons)	Crude Oil Production (Thousand	Crude oil import prices ($/barrel)	GDP PC ($)	GDP (Million $)	Currency (TL/$)	Inflation (%)
Population (Million persons)	1,000							
Crude Oil Production (Thousand	−0,154	−0,140						
Crude oil import prices ($/barrel)	0,728	0,715	−0,399					
GDP PC ($)	0,931	0,930	−0,016	0,692				
GDP (Million $)	0,912	0,911	−0,013	0,682	0,997			
Currency (TL/$)	0,788	0,786	−0,011	0,559	0,882	0,908		
Inflation (%)	−0,305	−0,289	0,310	−0,528	−0,403	−0,438	−0,485	
Household spending (Million $)	0,929	0,927	−0,065	0,713	0,993	0,996	0,907	−0,464
Temperature (C)	0,731	0,726	−0,140	0,571	0,758	0,761	0,695	−0,285
Wind Speed (m/sec)	0,022	0,025	−0,121	−0,029	−0,041	−0,053	−0,054	0,224
Air Pressure (hPa)	−0,034	−0,037	−0,066	0,022	−0,126	−0,138	−0,125	0,096
Relative Humidity (%)	−0,517	−0,504	0,321	−0,610	−0,530	−0,552	−0,579	0,580
Sunshine Time (Hr)	0,219	0,220	0,042	0,163	0,300	0,306	0,239	−0,111
Precipitation (Total, mm)	0,009	0,005	−0,169	0,022	−0,048	−0,064	−0,128	0,125
The proportion of Electricity Produced from Renewable Resources in Gross Electricity Consumption (%)	0,685	0,695	0,396	0,239	0,561	0,526	0,399	0,225
Variable	**Household** **spending** **(Million $)**	**Temperature (C)**	**Wind Speed** **(m/sec)**	**Air** **Pressure** **(hPa)**	**Relative** **Humidity** **(%)**	**Sunshine** **Time (Hr)**	**Precipitation** **(Total, mm)**	
Population (Million persons)								
Crude Oil Production (Thousand								
Crude oil import prices ($/barrel								
GDP PC ($)								
GDP (Million $)								
Currency (TL/$)								
Inflation (%)								
Household spending (Million $)								
Temperature (C)	0,771							
Wind Speed (m/sec)	−0,056	0,149						
Air Pressure (hPa)	−0,131	−0,162	0,153					
Relative Humidity (%)	−0,587	−0,398	0,126	0,238				
Sunshine Time (Hr)	0,292	0,380	−0,113	−0,065	0,075			
Precipitation (Total, mm)	−0,057	0,202	0,266	0,133	0,442	0,031		
Proportion) of Electricity Produced from Renewable Resources in Gross Electricity Consumption (%)	0,522	0,366	−0,001	0,062	−0,075	0,105	−0,044	

The correlation coefficient between GDP PC ($) and Population (Million persons) is 0.931, meaning there is a positive relationship. This indicates a strong positive relationship between gross domestic product per capita and population. Similarly, the correlation coefficient between GDP (Million $) and Population (Million persons) is 0.912, again indicating a positive relationship. In contrast, the correlation coefficient between Relative Humidity (%) and Population (Million persons) is −0.517, meaning there is a negative relationship. This indicates that there is a moderate negative relationship between relative humidity and population. The correlation table also includes two-way correlation coefficients, as well as values that show the strength and direction of correlations between variables. The correlation coefficient between Temperature (C) and Household spending (Million $) is 0.771, and the correlation coefficient between Household spending (Million $) and Temperature (C) is the same value. This indicates that the relationship between two variables is binary and one variable shows that it is not dependent on the other.

The correlation coefficient between the output variable Proportion of Electricity Produced from Renewable Resources in Gross Electricity Consumption (%) and Crude oil import prices ($/barrel) is 0.396. This indicates a positive relationship between oil import prices and the proportion of electricity produced from renewable sources. That is, as oil import prices increase, the proportion of electricity produced from renewable sources also tends to increase. On the other hand, the correlation coefficient between the variable “Proportion of Electricity Produced from Renewable Resources in Gross Electricity Consumption (%)” and “Relative Humidity (%)” is 0.580. This indicates a strong positive relationship between relative humidity and the proportion of electricity produced from renewable sources. That is, as the relative humidity increases, the proportion of electricity produced from renewable sources tends to increase. However, the correlation coefficient between the variable “Proportion of Electricity Produced from Renewable Resources in Gross Electricity Consumption (%)” and “Inflation (%)” is −0.485. This indicates a strong negative relationship between the inflation rate and the proportion of electricity produced from renewable sources. That is, as the inflation rate increases, the proportion of electricity produced from renewable sources tends to decrease. With these results, the reasons behind the differences in the relationships between input and output variables are more complex.

### 4.2 Prediction performance and ranking evaluation of ML algorithms

The study aims to examine the predictability of the Proportion of Electricity Produced from Renewable Resources in Gross Electricity Consumption (%), over 51 years, through three different ML algorithms. To analyze the performance of ML algorithms, 80% of the dataset was used for training and the remaining 20% for testing. The reliability of the prediction data obtained in each model was measured with four accuracy criteria: MAPE (%), MAE, RMSE, and MSE (%). Table 7 shows the performance measurement values of the ML algorithms run for the prediction process.

**Table 7:** Data on the prediction performances of ML algorithms.

Model	Phases	MSE	RMSE	MAE	MAPE	R^2^
RF	Test dataset	0.004	0.063	0.035	0.084	0.996
GB		0.137	0.370	0.120	0.240	0.856
AB		0.198	0.446	0.182	0.371	0.792
RF	Training dataset	0.141	0.375	0.240	0.376	0.881
GB		0.211	0.459	0.226	0.368	0.822
AB		0.024	0.154	0.100	0.260	0.980

RF, GB, and AB algorithms were evaluated on both test and training datasets. The RF model has the lowest MSE (0.004), RMSE (0.063), and MAE (0.035) values in the test data set. Moreover, the MAPE value (0.084) is also quite low, indicating high prediction accuracy according to the MAPE<10% criterion, while the R^2^ value (0.996) is also quite high, indicating that the model explains the data well. The GB model has slightly higher error values on the test dataset but still performs acceptable. The MAPE value (0.240) falls within the 10% < MAPE≤20% range, and the R^2^ value (0.856) is also quite high. Although the AB model has the highest MSE (0.198) and RMSE (0.446) values in the test dataset, the MAPE (0.371) value falls within the range of 20% < MAPE≤50%, which represents an acceptable prediction accuracy. Additionally, the R^2^ value (0.792) is also quite high.

On the training dataset, the performance of all three models is slightly lower, but they still provide acceptable prediction accuracy. While the RF model has the lowest error values in the training data set, the AB model has the lowest MAPE value (0.260). As a result, it is seen that all three models show an acceptable prediction performance and the RF model has the lowest error values. However, the TOPSIS method was used to carefully evaluate which model is best suited for a particular application, taking into account the strengths and weaknesses of each model.

TOPSIS method was used to rank the ML algorithms whose results were discussed above according to their performance. With this method, the performances of the algorithms are ranked by taking a systematic approach. Each performance measurement value of the ML algorithms was assigned as 0.2 (for each criterion to have an equal weight value, it should be equal to 1 in total), with equal weight. Table 8 shows the TOPSIS method ranking of the performance measurement values of ML algorithms.

**Table 8:** Ranking of ML algorithms.

Model	The normalized matrix					The weighted normalized matrix						
	MSE	RMSE	MAE	MAPE	R^2^	MSE	RMSE	MAE	MAPE	R^2^	Scores	Rank
RF	0.017	0.108	0.159	0.187	0.649	0.003	0.022	0.032	0.037	0.13	1.000	1
GB	0.569	0.635	0.543	0.534	0.558	0.114	0.127	0.109	0.107	0.112	0.349	2
AB	0.822	0.765	0.824	0.825	0.516	0.164	0.153	0.165	0.165	0.103	0.000	3
Positive	0.003	0.022	0.032	0.037	0.13	0.003	0.022	0.032	0.037	0.13	---	---
Negative	0.164	0.153	0.165	0.165	0.103	0.164	0.153	0.165	0.165	0.103	---	---

**Fig 5:**
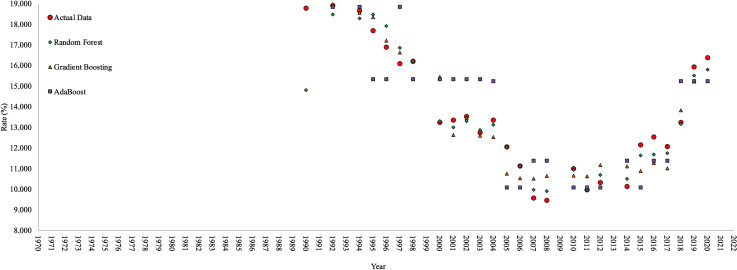
The comparison of selected data for prediction and actual data.

TOPSIS scores of RF, GB, and AB algorithms were calculated as 1.000, 0.349, and 0.000, respectively. The algorithm with the highest TOPSIS score is at the highest level of the ranking, and the prediction results obtained from this algorithm are close to the real data. The prediction data of all three algorithms are compared with the real data and shared in Fig 5.

The distribution of points is important to evaluate the similarity between actual and predicted values of each ML algorithm. The points of the RF algorithm follow the real data very closely. There is a strong relationship between the actual and predicted values of the RF algorithm. It is understood that the points belonging to the AB algorithm are randomly distributed and the predictions of this algorithm are incorrect in certain cases with the real values. As a result, it can be seen that the points are more densely located on the line for the RF model and there is more spread for the other models.

**Fig 6:**
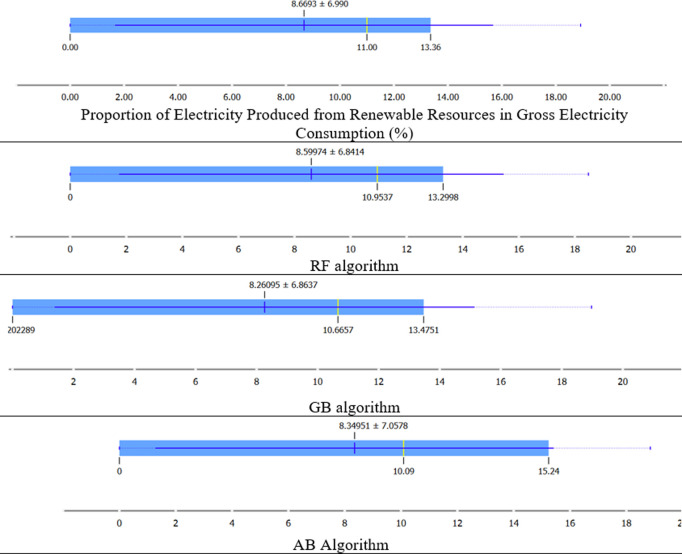
The box plots for data of Actual, RF, GB, and AB. a) Proportion of Electricity Produced from Renewable Resources in Gross Electricity Consumption (%). b) RF algorithm. c) GB algorithm. d) AB Algorithm.

Box plots were created to detect abnormalities such as outliers and the like between real data and predicted data obtained from ML algorithms. Fig 6 shows the box plots of the real, RF, AB, and GB algorithms. Quantity ranges of real and algorithm-based data were calculated and it was determined that they did not have outliers. In the box plot based on real data, the average value is calculated as 8.6693%. The standard deviation value calculated for this data is 6.99%. The average values of the RF, GB, and AB algorithms were calculated as 8.59974%, 8.26095%, and 8.34951%, respectively. The standard deviation values of these algorithms are calculated as 6.8414%, 6.8637%, and 7.0578%, respectively.

This study shows that ML algorithms perform well in predicting the electricity need obtained from renewable energy sources. To obtain prediction data, calculations were made by taking into account 12 parameters that are thought to be effective on the output variable. A solid structure was created by using the TOPSIS method to sort the algorithms that provide the prediction results. In addition, outlier data or abnormalities were detected by creating box plots of the data of the preferred algorithms to obtain prediction data. Thus, to ensure the reliability of the methods used for this study and the estimated results obtained, detailed variable types and different verification methods are included.

### 4.3 Discussion

In this study, ML algorithms RF, AB, and GB models were run to estimate Türkiye’s electricity consumption rate from renewable energy sources. In the study, oil, environmental, and economic input parameters that are effective in estimating the rate of electricity obtained from renewable energy sources were taken into account.

Renewable energy sources began to be used in 1990 to meet Türkiye’s electricity needs. However, in this study, data covering the years 1971–2021 were used to examine the data on the amount of energy obtained from renewable energy sources. These data were examined to see the impact of renewable energy sources on Türkiye’s electricity consumption and to see only the impact of forecast data on the rate of electricity usage from renewable energy types.

Factors used in the study include oil production, oil prices, weather parameters, and economic parameters. In particular, oil production and oil prices point to the strategic importance of electricity consumption based on renewable energy sources. The use of renewable energy sources reduces the energy sector’s dependence on oil demand while allowing it to meet energy supply in a more diverse, sustainable, and independent way. Weather parameters play a crucial role in forecasting weather-dependent renewable energy sources such as wind, hydropower, and solar energy. Therefore, parameters such as temperature, pressure, wind speed, humidity, sunshine duration, etc. have been preferred to estimate renewable energy production.

Economic parameters play a critical role in determining the impact of renewable energy projects in terms of electricity production. Renewable energy projects often require large-scale infrastructure investments, which affects the economic growth potential of countries. Successful implementation of these projects has the potential to create jobs, advance technology, and provide overall economic revival. For this reason, five different economic parameters were used in this study.

ML algorithms were used in this study to predict renewable energy use in Türkiye based on given factors. Algorithms such as RF, AB, and GB have been used in these predictions. These algorithms have been chosen for their ability to handle multiple variables, detect patterns in complex data, and provide accurate predictions. Four accuracy metrics such as R^2^, MAPE (%), MAE, RMSE, and MSE (%) were used to evaluate the performance of ML algorithms. According to the results obtained, it was seen that the RF model had the lowest error values and the highest prediction accuracy. Other models also provided acceptable prediction accuracy, but the RF model had the lowest error and highest accuracy. The GB model has slightly higher error values on the test dataset but still exhibits acceptable performance. The MAPE value (0.240%) shows a good prediction accuracy as it is in the range of 10% < MAPE≤20%. The R^2^ value (0.856%) is quite high and shows that the model explains the data well. The AB model, on the other hand, has the highest error values in the test data set, but still provides an acceptable prediction accuracy. The MAPE value (0.371%) indicates that the predictions are reasonably accurate as it is in the range of 20% < MAPE≤50%. Additionally, the R^2^ value (0.792%) is quite high, indicating that the model explains the data well.

In conclusion, this study examined the usability of different ML algorithms and the performances of these algorithms to predict renewable energy use in Türkiye. The results obtained show that ML algorithms can be used effectively in renewable energy forecasts and that these forecasts can play an important role in the formulation and implementation of energy policies.

This study has some limitations. First of all, the ratio of electricity production obtained from renewable energy sources to the electricity need was considered collectively from all renewable energy sources. The individual contributions of renewable energy resource types in electricity production have not been taken into account. While the amount of oil production and the cost per barrel of imported oil were taken into account in this study, data on the amount of imported oil was not used. Environmental factors were not taken into account regionally. There may be fluctuations in electricity consumption depending on different environmental parameters of the seven regions of the country taken into account for the study. In the study, prediction data for only three ML algorithms were obtained. In this study, these models were run using Orange version 3.34. There may be differences in the prediction results obtained by using models from other programs. Finally, electricity obtained from renewable energy sources has not been evaluated on a sectoral basis (industry, public, housing, transportation, commercial, etc.). For this reason, the proportional effects of the sectors’ electricity needs obtained from renewable energy sources have been ignored.

### 4.4 Policy implications and recommendations

The renewable energy sector is constantly evolving with the development of new and more efficient technologies. As Türkiye continues to invest in renewable energy, efficiency, and cost-effectiveness are expected to increase by adopting newer and more advanced technologies. This will make renewable energy more attractive to investors and further increase its use. A country’s economic conditions play an important role in the adoption of renewable energy. Türkiye’s economic growth and stability are of great importance in attracting investment in the renewable energy sector. However, any economic downturn or instability would cause a slowdown in the growth of renewable energy. Based on the projected growth in renewable energy in Türkiye, there are various policy implications and recommendations that the government should consider. First of all, the government needs to continue to implement supportive policies and incentives that will attract investment in the renewable energy sector. This could include tax breaks, subsidies, and feed-in tariffs for renewable energy producers. Second, the focus should be on diversifying Türkiye’s renewable energy mix. As a result, Türkiye has made significant progress in the use of renewable energy and this trend is expected to continue in the future. But to ensure sustainable growth, the government needs to continue to implement supportive policies and diversify the country’s renewable energy mix.

## 5. Conclusion and further reading

As the electricity demand is constant, the sources of electricity are constantly changing and evolving. Nowadays, electricity use is rising to meet the energy demands of the technological devices in use and population growth. Countries are compelled to increase and diversify their electricity-generating resources to meet this demand. With the emergence of renewable energy sources, countries have started to meet their electricity needs. Countries have increasingly turned to renewable energy sources for electricity generation due to the negative environmental effects and unsustainability of traditional sources such as oil and coal. Today, there is a trend towards wind and solar energy types. This study considers Türkiye’s electricity needs from all renewable energy sources, including wind and solar. Creating a suitable model to predict future electricity needs, particularly from renewable energy sources, is crucial. This study provides a comprehensive comparison of machine learning algorithms to estimate the amount of electricity generated from renewable energy sources, considering Türkiye’s population, weather parameters, economic indicators, and oil supply. The main findings of the study are as follows:

- With the electricity needs being met from renewable energy sources, the importance of environmental, economic, and oil resources on renewable energy sources has emerged both statistically and in forecasting.
- A forecast model for the electricity needs obtained from renewable energy sources was developed for future periods and contributed to the formation of a strategic plan in the field of energy.
- The RF model outperformed the AB and GB models in predicting electricity needs from renewable energy sources.
- Türkiye’s tendency towards renewable energy sources is increasing day by day to provide a sustainable electricity supply.
- It has been emphasized that sustainable energy sources for the constantly needed electricity create both positive environmental effects and safe energy supply areas.

As a result, this study discussed a method that allows estimating the needs of future periods, emphasizing that it is a sustainable system by considering the electricity needs to be met from renewable energy sources. Especially since traditional energy sources used in electricity generation have negative environmental effects and are not sustainable, countries are directed to the renewable energy sector. Especially countries that do not have enough traditional energy resources become dependent on foreign sources for electricity production and are negatively affected economically. In addition to all these, this study can be an important guide in contributing to the energy policies of countries that take into account the electricity needs of future periods and in the formation of strategic plans for energy maintenance.

## Supporting information

S1 Data Raw data including population, economic, climate, and energy variables from 1971 to 2021. (XLSX)

## References

[1] Holdren JP. The energy innovation imperative: addressing oil dependence, climate change, and other 21st century energy challenges. Innov Technol Gov Glob. 2006;1(2):3–23. doi: 10.1162/itgg.2006.1.2.3

[2] Arutyunov VS, Lisichkin GV. Energy resources of the 21st century: problems and forecasts. Can renewable energy sources replace fossil fuels? Russ Chem Rev. 2017;86(8):777–804. doi: 10.1070/rcr4723

[3] Pao H-T, Fu H-C. Renewable energy, non-renewable energy and economic growth in Brazil. Renew Sustain Energy Rev. 2013;25:381–92. doi: 10.1016/j.rser.2013.05.004

[4] Scheffran J, Felkers M, Froese R. Economic growth and the global energy demand. In: Green energy to sustainability: strategies for global industries; 2020. p. 1–44.

[5] Kalair A, Abas N, Saleem MS, Kalair AR, Khan N. Role of energy storage systems in energy transition from fossil fuels to renewables. Energy Storage. 2021;3:e135.

[6] Al-Maamary HMS, Kazem HA, Chaichan MT. The impact of oil price fluctuations on common renewable energies in GCC countries. Renew Sustain Energy Rev. 2017;75:989–1007. doi: 10.1016/j.rser.2016.11.079

[7] Erdoğan S, Gedikli A, Çevik Eİ, Erdoğan F, Çevik E. Precious metals as safe-haven for clean energy stock investment: evidence from nonparametric Granger causality in distribution test. Resour Policy. 2022;79:102945. doi: 10.1016/j.resourpol.2022.102945

[8] Griffiths S. A review and assessment of energy policy in the Middle East and North Africa region. Energy Policy. 2017;102:249–69. doi: 10.1016/j.enpol.2016.12.023

[9] Abbasi KR, Shahbaz M, Zhang J, Irfan M, Alvarado R. Analyze the environmental sustainability factors of China: the role of fossil fuel energy and renewable energy. Renew Energy. 2022;187:390–402. doi: 10.1016/j.renene.2022.01.066

[10] Ayaz Atalan Y, Tayanç M, Erkan K, Atalan A. Development of nonlinear optimization models for wind power plants using box-behnken design of experiment: a case study for Turkey. Sustainability. 2020;12(15):6017. doi: 10.3390/su12156017

[11] Ezbakhe F, Pérez-Foguet A. Decision analysis for sustainable development: the case of renewable energy planning under uncertainty. Eur J Oper Res. 2021;291(2):601–13. doi: 10.1016/j.ejor.2020.02.037

[12] Evans A, Strezov V, Evans TJ. Assessment of sustainability indicators for renewable energy technologies. Renew Sustain Energy Rev. 2009;13(5):1082–8. doi: 10.1016/j.rser.2008.03.008

[13] Atalan YA, Atalan A. Integration of the machine learning algorithms and I-MR statistical process control for solar energy. Sustainability. 2023;15(18):13782. doi: 10.3390/su151813782

[14] Wen S, Jia Z. The energy, environment and economy impact of coal resource tax, renewable investment, and total factor productivity growth. Resour Policy. 2022;77:102742. doi: 10.1016/j.resourpol.2022.102742

[15] Chang L, Saydaliev HB, Meo MS, Mohsin M. How renewable energy matter for environmental sustainability: evidence from top-10 wind energy consumer countries of European Union. Sustain Energy Grids Netw. 2022;31:100716. doi: 10.1016/j.segan.2022.100716

[16] Asif M, Muneer T. Energy supply, its demand and security issues for developed and emerging economies. Renew Sustain Energy Rev. 2007;11(7):1388–413. doi: 10.1016/j.rser.2005.12.004

[17] Brouwer AS, van den Broek M, Seebregts A, Faaij A. Impacts of large-scale Intermittent Renewable Energy Sources on electricity systems, and how these can be modeled. Renew Sustain Energy Rev. 2014;33:443–66. doi: 10.1016/j.rser.2014.01.076

[18] Adão B, Narajabad B, Temzelides T. Renewable technology adoption costs and economic growth. Energy Econ. 2024;129:107255. doi: 10.1016/j.eneco.2023.107255

[19] Timmons D, Harris JM, Roach B. The economics of renewable energy, Vol. 52. Global Development And Environment Institute, Tufts University; 2014. p. 1–52.

[20] Shen Y-C, Lin GTR, Li K-P, Yuan BJC. An assessment of exploiting renewable energy sources with concerns of policy and technology. Energy Policy. 2010;38(8):4604–16. doi: 10.1016/j.enpol.2010.04.016

[21] Ahmad T, Madonski R, Zhang D, Huang C, Mujeeb A. Data-driven probabilistic machine learning in sustainable smart energy/smart energy systems: key developments, challenges, and future research opportunities in the context of smart grid paradigm. Renew Sustain Energy Rev. 2022;160:112128. doi: 10.1016/j.rser.2022.112128

[22] Vennila C, Titus A, Sudha TS, Sreenivasulu U, Reddy NPR, Jamal K, et al. Forecasting solar energy production using machine learning. Int J Photoenergy. 2022;2022:1–7. doi: 10.1155/2022/7797488

[23] Rahman MM, Shakeri M, Tiong SK, Khatun F, Amin N, Pasupuleti J, et al. Prospective methodologies in hybrid renewable energy systems for energy prediction using artificial neural networks. Sustainability. 2021;13(4):2393. doi: 10.3390/su13042393

[24] Aslam S, Herodotou H, Mohsin SM, Javaid N, Ashraf N, Aslam S. A survey on deep learning methods for power load and renewable energy forecasting in smart microgrids. Renew Sustain Energy Rev. 2021;144:110992. doi: 10.1016/j.rser.2021.110992

[25] Li J, Ward JK, Tong J, Collins L, Platt G. Machine learning for solar irradiance forecasting of photovoltaic system. Renew Energy. 2016;90:542–53. doi: 10.1016/j.renene.2015.12.069

[26] Khan GM, Ali J, Mahmud SA. Wind power forecasting — An application of machine learning in renewable energy. 2014 International Joint Conference on Neural Networks (IJCNN); 2014. p. 1130–7. doi: 10.1109/IJCNN.2014.6889771

[27] Zulkifly Z, Baharin KA, Gan CKIM. Improved machine learning model selection techniques for solar energy forecasting applications. IJRER. 2021;11:308–19.

[28] Sharifzadeh M, Sikinioti-Lock A, Shah N. Machine-learning methods for integrated renewable power generation: a comparative study of artificial neural networks, support vector regression, and Gaussian Process Regression. Renew Sustain Energy Rev. 2019;108:513–38. doi: 10.1016/j.rser.2019.03.040

[29] Martín L, Zarzalejo LF, Polo J, Navarro A, Marchante R, Cony M. Prediction of global solar irradiance based on time series analysis: application to solar thermal power plants energy production planning. Solar Energy. 2010;84(10):1772–81. doi: 10.1016/j.solener.2010.07.002

[30] Fernandez-Jimenez LA, Muñoz-Jimenez A, Falces A, Mendoza-Villena M, Garcia-Garrido E, Lara-Santillan PM, et al. Short-term power forecasting system for photovoltaic plants. Renew Energy. 2012;44:311–7. doi: 10.1016/j.renene.2012.01.108

[31] Heinermann J, Kramer O. Machine learning ensembles for wind power prediction. Renew Energy. 2016;89:671–9. doi: 10.1016/j.renene.2015.11.073

[32] Demolli H, Dokuz AS, Ecemis A, Gokcek M. Wind power forecasting based on daily wind speed data using machine learning algorithms. Energy Convers Manag. 2019;198:111823. doi: 10.1016/j.enconman.2019.111823

[33] Carneiro TC, Rocha PAC, Carvalho PCM, Fernández-Ramírez LM. Ridge regression ensemble of machine learning models applied to solar and wind forecasting in Brazil and Spain. Appl Energy. 2022;314:118936. doi: 10.1016/j.apenergy.2022.118936

[34] Salazar-Caceres F, Ramirez-Murillo H, Torres-Pinzón CA, Camargo-Martínez MP. Performance estimation technique for solar-wind hybrid systems: a machine learning approach. Alex Eng J. 2024;87:175–85. doi: 10.1016/j.aej.2023.12.029

[35] Eshchanov BR, Grinwis Plaat Stultjes M, Eshchanov RA, Salaev SK. Prospects of renewable energy penetration in Uzbekistan—Perception of the Khorezmian people. Renew Sustain Energy Rev. 2013;21:789–97. doi: 10.1016/j.rser.2013.01.023

[36] Wang H, Lei Z, Zhang X, Zhou B, Peng J. A review of deep learning for renewable energy forecasting. Energy Convers Manag. 2019;198:111799. doi: 10.1016/j.enconman.2019.111799

[37] Kaygusuz K, Kaygusuz A. Renewable energy and sustainable development in Turkey. Renew Energy. 2002;25(3):431–53. doi: 10.1016/s0960-1481(01)00075-1

[38] Boghetti R, Fantozzi F, Kämpf JH, Salvadori G. Understanding the performance gap: a machine learning approach on residential buildings in Turin, Italy. J Phys: Conf Ser. 2019;1343(1):012042. doi: 10.1088/1742-6596/1343/1/012042

[39] Abdmouleh Z, Alammari RAM, Gastli A. Review of policies encouraging renewable energy integration & best practices. Renew Sustain Energy Rev. 2015;45:249–62. doi: 10.1016/j.rser.2015.01.035

[40] Sawin JL. National policy instruments: Policy lessons for the advancement and diffusion of renewable energy technologies around the world. In: Renewable energy. Routledge; 2012. p. 71–114.

[41] Güner YE. The development of the Turkish power market with special respect to renewable power generation in Turkey. Dissertation. Clausthal-Zellerfeld: Technische Universität Clausthal, 2017; 2017.

[42] Akca Prill M. Energy dependency, the potential supply of renewable energies and the political responses in Turkey in the decades since the oil-crisis. Dissertation. Darmstadt: Technische Universität Darmstadt, 2019; 2020.

[43] Celik AN. Analysis of energy supply, installed power and renewable capacity in the world, the EU and Turkey. Düzce Üniversitesi Bilim ve Teknoloji Dergisi. 2021;9(3):500–19. doi: 10.29130/dubited.827250

[44] Hereher M, El Kenawy AM. Exploring the potential of solar, tidal, and wind energy resources in Oman using an integrated climatic-socioeconomic approach. Renew Energy. 2020;161:662–75. doi: 10.1016/j.renene.2020.07.144

[45] Sharif A, Baris-Tuzemen O, Uzuner G, Ozturk I, Sinha A. Revisiting the role of renewable and non-renewable energy consumption on Turkey’s ecological footprint: evidence from Quantile ARDL approach. Sustain Cities Soc. 2020;57:102138. doi: 10.1016/j.scs.2020.102138

[46] Nwozor A, Oshewolo S, Owoeye G, Okidu O. Nigeria’s quest for alternative clean energy development: a cobweb of opportunities, pitfalls and multiple dilemmas. Energy Policy. 2021;149:112070. doi: 10.1016/j.enpol.2020.112070

[47] Kaygusuz O, Ayhan T, Kaygusuz K. Renewable energy for low carbon economy and sustainable development in Turkey. J Eng Res Appl Sci. 2021;10:1717–29.

[48] Konurhan Z, Yücesan M, Gül M. An integrated Bayesian Best-Worst Method and GIS-based approach for offshore wind power plant site selection: a case study in North Aegean and Marmara Sea (Türkiye). Türk Coğrafya Dergisi. 2023;(82):7–22. doi: 10.17211/tcd.1214671

[49] Al-Shetwi AQ. Sustainable development of renewable energy integrated power sector: trends, environmental impacts, and recent challenges. Sci Total Environ. 2022;822:153645. doi: 10.1016/j.scitotenv.2022.153645 35124039

[50] Kapluhan E. Evaluation of Turkey’s renewable energy potential in terms of 2023 energy vision. Вестник Евразийского национального университета имени ЛН Гумилева Серия: Химия География Экология. 2021;135:71–87.

[51] Erat S, Telli A, Ozkendir OM, Demir B. Turkey’s energy transition from fossil-based to renewable up to 2030: milestones, challenges and opportunities. Clean Techn Environ Policy. 2020;23(2):401–12. doi: 10.1007/s10098-020-01949-1

[52] Ediger VS, Akar S. Historical pattern analysis of global geothermal power capacity development. Golden, CO (United States): National Renewable Energy Laboratory (NREL); 2023.

[53] OECD Data. Crude oil import prices; 2023.

[54] TSMS. Turkish state meteorological service; 2023. Available from: https://www.mgm.gov.tr/eng/forecast-cities.aspx

[55] OECD Data. Economy; 2023.

[56] OECD Data. Renewable energy; 2023.

[57] Sahin EK. Assessing the predictive capability of ensemble tree methods for landslide susceptibility mapping using XGBoost, gradient boosting machine, and random forest. SN Appl Sci. 2020;2(7):1308. doi: 10.1007/s42452-020-3060-1

[58] Bernard S, Heutte L, Adam S. On the selection of decision trees in Random Forests. 2009 International Joint Conference on Neural Networks. IEEE; 2009. p. 302–7. doi: 10.1109/ijcnn.2009.5178693

[59] Atalan A, Şahin H, Atalan YA. Integration of machine learning algorithms and discrete-event simulation for the cost of healthcare resources. Healthcare (Basel). 2022;10(10):1920. doi: 10.3390/healthcare10101920 36292372 PMC9601943

[60] Paul D, Su R, Romain M, Sébastien V, Pierre V, Isabelle G. Feature selection for outcome prediction in oesophageal cancer using genetic algorithm and random forest classifier. Comput Med Imaging Graph. 2017;60:42–9. doi: 10.1016/j.compmedimag.2016.12.002 28087102

[61] Barashid K, Munshi A, Alhindi A. Wind farm power prediction considering layout and wake effect: case study of Saudi Arabia. Energies. 2023;16(2):938. doi: 10.3390/en16020938

[62] Saleem F, Ullah Z, Fakieh B, Kateb F. Intelligent decision support system for predicting student’s e-learning performance using ensemble machine learning. Mathematics. 2021;9(17):2078. doi: 10.3390/math9172078

[63] Tanha J, Abdi Y, Samadi N, Razzaghi N, Asadpour M. Boosting methods for multi-class imbalanced data classification: an experimental review. J Big Data. 2020;7(1):1–47. doi: 10.1186/s40537-020-00349-y

[64] Wang L-L, Ngan HYT, Yung NHC. Automatic incident classification for large-scale traffic data by adaptive boosting SVM. Inf Sci. 2018;467:59–73. doi: 10.1016/j.ins.2018.07.044

[65] Guelman L. Gradient boosting trees for auto insurance loss cost modeling and prediction. Expert Syst Appl. 2012;39(3):3659–67. doi: 10.1016/j.eswa.2011.09.058

[66] Wang Y, Chang L, Mao M, Hatziargyriou ND. Non-intrusive load decomposition based on SAMME.R-DT algorithm. 2019 IEEE 10th International Symposium on Power Electronics for Distributed Generation Systems (PEDG); 2019. p. 515–9. doi: 10.1109/pedg.2019.8807513

[67] González S, García S, Del Ser J, Rokach L, Herrera F. A practical tutorial on bagging and boosting based ensembles for machine learning: algorithms, software tools, performance study, practical perspectives and opportunities. Inf Fusion. 2020;64:205–37. doi: 10.1016/j.inffus.2020.07.007

[68] Ceylan Z. The impact of COVID-19 on the electricity demand: a case study for Turkey. Int J Energy Res. 2021;45(9):13022–39. doi: 10.1002/er.6631 34230753 PMC8250713

[69] Chakraborty S. TOPSIS and modified TOPSIS: a comparative analysis. Decis Anal J. 2022;2:100021. doi: 10.1016/j.dajour.2021.100021

[70] Yoon K. Multiple attributes decision making methods and applications. Nowy Jork: Wydawnictwo Springer-Verlag; 1981.

[71] Chakraborty S, Mandal A. A novel TOPSIS based consensus technique for multiattribute group decision making. 2018 18th International Symposium on Communications and Information Technologies (ISCIT); 2018. p. 322–6. doi: 10.1109/iscit.2018.8587952

[72] Chakraborty S, Yeh C-H. Comparison based group ranking outcome for multiattribute group decisions. 2012 UKSim 14th International Conference on Computer Modelling and Simulation. IEEE; 2012. p. 324–7. doi: 10.1109/uksim.2012.53

[73] Yeh C. The selection of multiattribute decision making methods for scholarship student selection. Int J Selection Assess 2003;11(4):289–96. doi: 10.1111/j.0965-075x.2003.00252.x

[74] Yoon KP, Hwang CL. Multiple attribute decision making: an introduction. Sage Publications; 1995.

